# Supplementary figures and images for: Transcriptomics Integrated with Metabolomics Unveil Carotenoids Accumulation and Correlated Gene Regulation in White and Yellow-Fleshed Turnip (Brassica rapa ssp. rapa)
Source: Genes (Basel). 2022 May 26;13(6):953. doi: 10.3390/genes13060953 (PMC9222417; doi:10.3390/genes13060953)

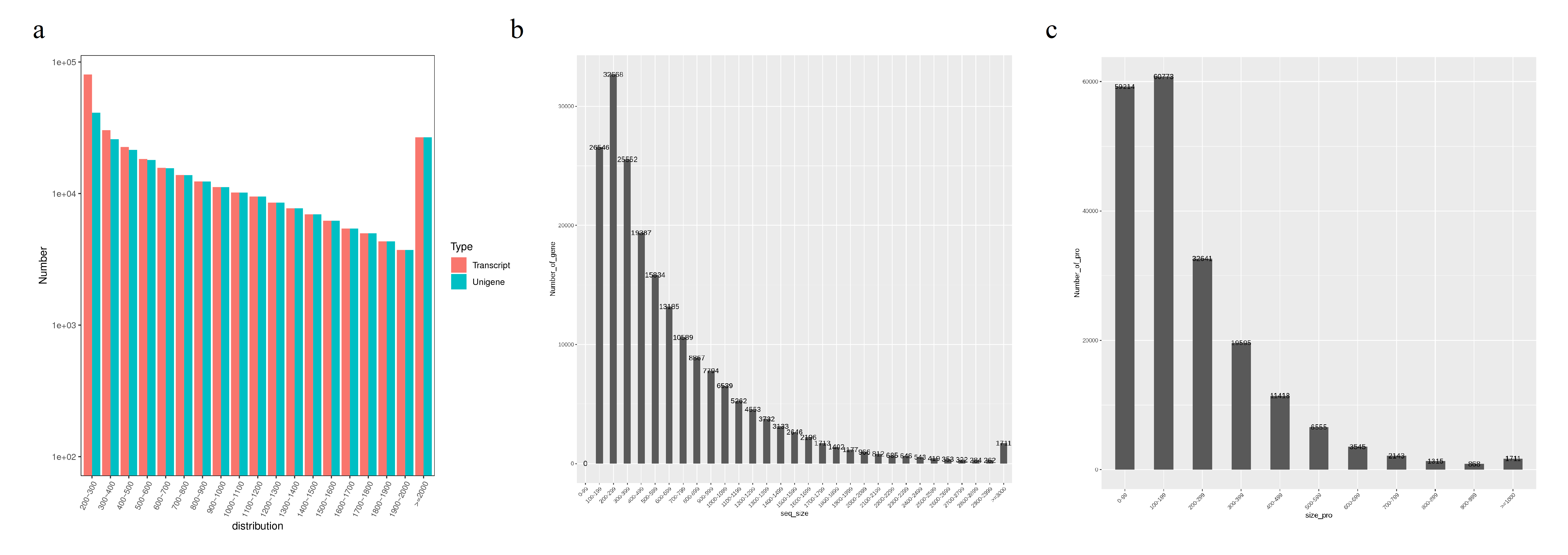

Supplement: Supplementary file 1 [file genes-13-00953-s001.zip › ╕╜═╝▒φ/Figure S1.png]
